# Cadaveric and Ultrasound Validation of Percutaneous Electrolysis Approach at the Achilles Tendon as a Potential Treatment for Achilles Tendinopathy: A Pilot Study

**DOI:** 10.3390/ijerph191911906

**Published:** 2022-09-21

**Authors:** Laura Calderón-Díez, José Luis Sánchez-Sánchez, Miguel Robles-García, Pedro Belón-Pérez, César Fernández-de-las-Peñas

**Affiliations:** 1 Department of Physical Therapy, Universidad de Salamanca, 37008 Salamanca, Spain; 2 Department of Anatomy and Histology, Faculty of Medicine, Universidad de Salamanca, 37008 Salamanca, Spain; 3 Real Madrid C.F., 28055 Madrid, Spain; 4 Department of Physical Therapy, Occupational Therapy, Physical Medicine and Rehabilitation, Universidad Rey Juan Carlos (URJC), 28922 Alcorcón, Spain; 5 Cátedra Institucional en Docencia, Clínica e Investigación en Fisioterapia: Terapia Manual, Punción Seca y Ejercicio Terapéutico, Universidad Rey Juan Carlos, 28922 Alcorcón, Spain

**Keywords:** Achilles tendon, cadaver, sural nerve, tendinopathy, percutaneous electrolysis

## Abstract

Achilles tendon tendinopathy (AT) is a musculoskeletal condition characterized by pain in the Achilles tendon and impaired physical performance or sport activities. AT is difficult to treat, and the results are variable. Preliminary evidence suggests a positive effect for pain of percutaneous electrolysis in patients with tendinopathy. Our aim was to determine the validity and safety of a percutaneous electrolysis approach targeting the interphase between the Achilles tendon and the Kager’s fat with ultrasound imaging in both healthy individuals and on a fresh cadaver model (not ultrasound guiding). A needle was inserted from the medial to the lateral side under the body of the Achilles tendon, just between the tendon and the Kager’s triangle, about 5 cm from the insertion of tendon in the calcaneus in 10 healthy volunteers (ultrasound study) and 10 fresh cadaver legs. An accurate needle penetration of the interphase was observed in 100% of the approaches, in both human and cadaveric models. No neurovascular bundle of the sural nerve was pierced in any insertion. The distance from the tip of the needle to the sural nerve was 5.28 ± 0.7 mms in the cadavers and 4.95 ± 0.68 mms in the volunteer subjects, measured in both cases at a distance of 5 cm from the insertion of the Achilles tendon. The results of the current study support that percutaneous electrolysis can be safely performed at the Kager’s fat–Achilles tendon interphase if it is US guided. In fact, penetration of the sural nerve was not observed in any needle approach when percutaneous needling electrolysis was performed by an experienced clinician. Future studies investigating the clinical effectiveness of the proposed intervention are needed.

## 1. Introduction

According to current theories, tendinopathies are the consequence of the response of the failed tendon healing, showing hypervascularization, alterations in the tendon structure with degeneration and aleatory proliferation of tenocytes, and alteration of fibers of collagen and of the extracellular matrix [1]. However, the origin of tendon-related pain is still controversial. The abnormal presence of neovascularization in some tendinopathies is accompanied by a neoinnervation. Nowadays, it is believed that these neo nerves can play a central role in the development of tendon-related pain [2,3].

Achilles tendon tendinopathy (AT) is a musculoskeletal condition characterized by pain in the Achilles tendon and impaired physical performance or sport activities. AT can be classified as insertional and non-insertional (affecting the body of the tendon), two disorders with different subjacent pathophysiological processes and with different treatment approaches [4]. AT is more common in athletes and represents from 6% to 17% of running injuries; however, it also appears in non-athletic patients without history of an increase of activity [5]. That is why the etiology of AT continues to be an object of debate and is likely to be caused by intrinsic and extrinsic factors [6].

AT is difficult to treat, and the results are variable [7]. The initial handling is like the rest of tendinopathies. The first-line treatment of AT is conservative management with physical therapy based fundamentally in controlling the tendon load [7,8]. Other therapies as shock waves or infiltrations also are used [8,9,10,11], but their results are variable and there is no treatment considered gold standard for AT management. The vast treatments commonly used by these patients lack evidence-based support, and patients undergo treatment with unpredictable clinical response [10,11].

Non-surgical treatments are the mainstay of the AT; however, in 25% the conservative option fails, patients continue with pain and require surgery. It is recommended not to perform surgery before 6 months of failing conservative treatment [12]. Traditional open surgical approaches for handling both AT and the rupture of Achilles tendon result in high risk of infections and morbidity [13]. The widening exposure that provides open processes can cause an extensive iatrogenic alteration in the subcutaneous tissues and paratenon, which increases the potential of peritendinous adhesions [14,15,16].

For this reason, during the last years, minimally invasive surgical approaches have been developed for the management of AT, with the aim of minimizing the damage of soft tissues and allowing for faster rehabilitation and recovery. In the case of tendinopathies in the Achilles tendon body, in 2009, Longo and Mafulli developed a minimally invasive surgical process intrinsically different to the classic one in use today [17]. This technique does not intend to approach directly to the pathological lesion focus; otherwise, its aim is, through minimum lateral incisions to the tendon and making a scraping, removing neural tissues present in the interphase between the Achilles tendon and Kager’s fat looking for a denervation of the area and with it an improvement in pain. This technique of stripping has been described as low morbidity and low cost, and some data show good results in patient with AT in the tendon body [18]. However, both open and minimally invasive surgical procedures also present complications. One of them is the iatrogenic injury of the sural nerve, a sensitive nerve with a close relation with Achilles tendon, in which neuropraxias have been reported [19].

In the last years, minimally invasive techniques, such as percutaneous electrolysis and dry needling, have shown to be potentially effective for tendon-related pain. Percutaneous electrolysis consists of the application of a galvanic electric current throughout a solid filament needle. Current evidence supports positive results of this technique in terms of pain and functionality [20,21,22]. A meta-analysis found moderate evidence suggesting a positive effect of percutaneous electrolysis, when ultrasound guided, for pain and disability in patients with tendon-related pain [20]. Percutaneous electrolysis is clinically applied to different tendinopathies, including AT. So far, no previous study has examined the adverse effects associated with the application of this invasive procedure on the AT in physiotherapy. Established criteria of uniformity in terms of the methodology, course, and relationship of the Achilles tendon with surrounding structures will allow for more safe invasive procedures of physiotherapy to be applied.

The aim of this pilot study was to determine the validity and safety of percutaneous electrolysis approach targeting the interphase of the body of the Achilles tendon in both human (ultrasound-guided) and in fresh cadaver (not ultrasound-guided) models.

## 2. Methods

### 2.1. Procedure

Ten healthy volunteers participated in the ultrasound (US)-guided study, whereas ten lower limbs of fresh cadavers donated to the department of anatomy and histology of the University of Salamanca (Spain) were used for the cadaveric part. Healthy volunteers were included if they presented no history of musculoskeletal pain in the lower extremity the previous two years, no medical comorbidity provoking pain symptoms such as fibromyalgia syndrome or arthritis rheumatoid, and no previous surgery in the lower extremity. Cadavers were checked for the presence of any structural abnormalities that could influence the anatomical study. The procedure involving healthy participants was conducted following the Declaration of Helsinki and it was approved by the Human Research Ethics Committee (CBE) of the University of Salamanca (USAL 2021/550), Spain. Participants signed a written informed consent form prior to inclusion.

### 2.2. Anatomical Procedure on Fresh Cadaver

**Figure 1:**
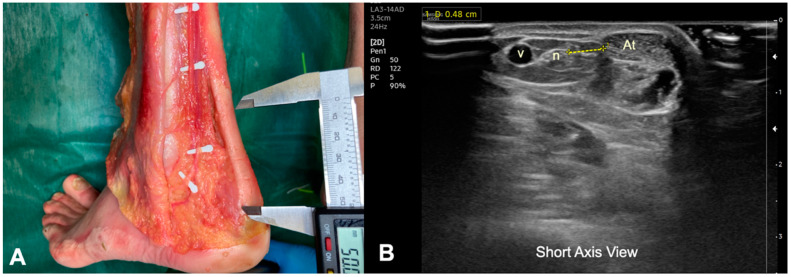
(**A**) Anatomical scheme of the course of the sural nerve and its relationship with the Achilles tendon on the lateral aspect of the ankle (white pins). Measurement in cadaver at 5 cm from the insertion in the calcaneus. (**B**) Ultrasound image (short axis view) of the relationship and measurement of the sural nerve with the lateral border of the Achilles tendon in a healthy volunteer. (At)—Achilles Tendon, (n)—Sural nerve, (v)—Lesser saphenous vein.

**Figure 2:**
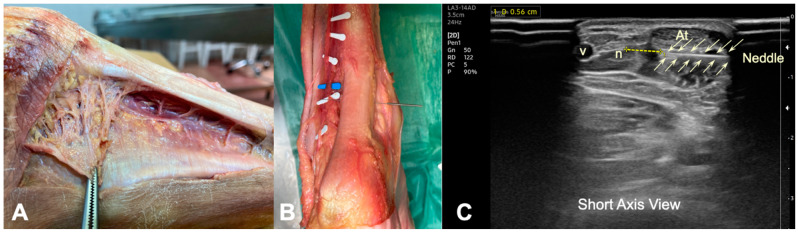
(**A**) Cadaveric view of the relationship between Kager’s fat and the deep face of the Achilles tendon. (**B**) Cadaveric detailed view of the needling insertion at the interphase between the Achilles tendon and Kager’s fat at 5 cm from the insertion in the calcaneus. (**C**) Ultrasound image (short axis view) of the measurement from the tip of the needle to the sural nerve inserted at the Kager’s fat–Achilles tendon interphase (from medial border) measured 5 cm from the insertion in the calcaneus. (At)—Achilles Tendon, (n)—Sural nerve, (v)—lesser saphenous vein.

A sample of five corpses (bilateral) was used and dissection of the posterior region of the foot was performed. The low extremity was kept with the knee extended and the foot was placed at 15° of plantar flexion. The legs were dissected in the long axis, longitudinally from the popliteal fossa to the foot, and skin and subcutaneous fascia tissue of the dorsal aspect of the leg were removed. This allowed for the visualization of the gastrocnemius muscles and the Achilles tendon until its insertion into the calcaneus. The Kager’s triangle, the lesser saphenous vein, and the sural nerve were also identified (Figure 1A and Figure 2A).

The needle was inserted into the cadaver about 5 cm from the insertion of Achilles tendon in the calcaneus, from medial to lateral, at the interphase between the tendon and the Kager’s fat. It was left in situ during the anatomical dissection, and the distance between the lateral border of the tendon and sural nerve was measured with a digital caliper (Electro DH Mod. 60.210). The cadaveric study was performed without US guiding (Figure 2B).

### 2.3. Percutaneous Electrolysis Procedures

The US-guided electrolysis percutaneous intervention targeting the body of the Achilles tendon was performed in 10 healthy volunteers. The intervention was US guided by using an HS50 Samsumg^®^ device equipped with an 14 MhZ superficial linear transducer (LA3-14AD). The procedures were performed by a physical therapist with 15 years of experience in musculoskeletal US-guided needling interventions. The depth of US assessment was fixed at 3.5 cm in both healthy volunteers and cadavers for standardization of the procedure. This depth was chosen based on the clinical experience of the assessor with the aim to properly visualize all structures. In the short axis, the sural nerve was located next to the lesser saphenous vein. In that position, the common branch of the sural nerve was identified as an oval structure that was relatively hypoechoic, surrounded by a rim of hyperechoic connective tissue anterior to the vein and lateral to Achilles tendon (Figure 1B).

**Figure 3:**
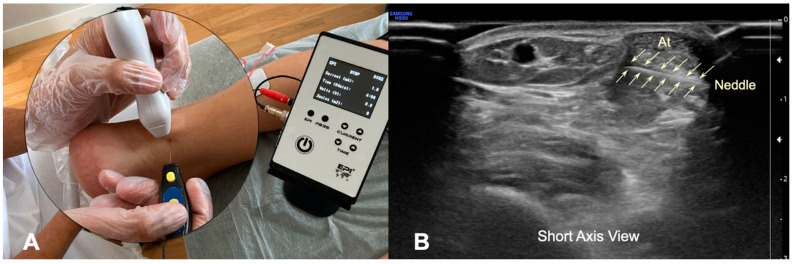
(**A**) Illustration of the percutaneous electrolysis approach at the Kager’s fat–Achilles tendon interphase (from medial border). (**B**) Ultrasound imaging of the needle reaching the interphase of the Kager’s fat.

The approach to the Achilles tendon was performance at the height of the body of the tendon, 5 cm from its insertion in the calcaneus, because it is the area where the degenerative focus settles most frequently in AT. In a prone position, a 25 × 0.3 mm filiform solid needle (AguPunt, Barcelona, Spain) was inserted from the medial side to the lateral side under the body of the tendon, just between the tendon and the Kager’s triangle (Figure 2A). This approach was US guided by the clinician to correctly reach the fat–tendon interphase (below) (Figure 2A,B). Several routes were made with needles at 30°, 45°, and 70° degrees approximately from the first approach, both caudally and cranially, but always in the interphase, without puncturing the Achilles tendon. In addition, the distance between the lateral border of the tendon and the sural nerve was sonographically measured (Figure 1B and Figure 3B).

### 2.4. Statistical Analysis

The statistical analysis was conducted with the SPSS statistical package (25.0 Version). Data are presented as means ± standard deviations. Independent Student’s *t*-tests were used to determine differences in the distance to the sural nerve between cadaver specimens and healthy subjects and between males and females. The statistical significance was set at a value of *p* < 0.05.

## 3. Results

This study included 10 healthy volunteers (6 females, mean age: 45 ± 14 years) and 10 legs of cadaver specimens (2 females, mean age: 70 ± 11 years). The application of percutaneous electrolysis targeting the interphase between the Achilles tendon and Kager’s triangle was possible in all participants (accuracy 100%). Neither the sural nerve nor the lesser saphenous vein was pierced with the needle (Figure 2).

We observed that the relation between the Achilles tendon and the sural nerve is close. In all subjects, the sural nerve ran at the level of distal third of the leg subcutaneously parallel to the lesser saphenous vein (Figure 1A,B). However, we observed an anatomical variation where the nerve ran medial to the vein in 20% of our sample. The mean distance from the Achilles tendon to the sural nerve was similar (*p* = 0.268) between the cadavers (mean: 5.28 ± 0.07 mms) (Figure 1A) and healthy subjects (mean: 4.95 ± 0.07 mms) (Figure 1B), measured in both cases at a distance of 5 cm from the insertion of the Achilles tendon. In any of the insertions, neither the sural nerve nor the lesser saphenous vein was pierced by the needle; therefore, we can consider a safer approach performed by ultrasonic control (Figure 3B).

A preliminary sex analysis did not reveal significant differences in the distance from the Achilles tendon to the sural nerve (*p* = 0.726) between males (mean: 5.15 ± 0.06 mms) and females (mean: 5.05 ± 0.07 mms).

## 4. Discussion

This study revealed that the application of US-guided percutaneous electrolysis targeting the interphase between the Achilles tendon and the Kager’s fat can be considered a safe procedure since the needle did not pierce the sural nerve or the lesser saphenous vein.

The sural nerve has a close anatomic relationship with the lateral border of Achilles tendon and by its proximity is at risk during some procedures on the tendon. Iatrogenic injuries of the sural nerve are described as a frequent complication in surgical procedures in the treatment of AT, with 14% of cases in percutaneous tendon repairs [23] and 19% in minimally invasive surgeries [24]. Several studies suggest intraoperative ultrasound assistance in percutaneous treatment of Achilles tendon injuries, considering it an effective and reliable method to minimize the risk of nerve injury [25,26].

Cadaveric studies allow for the development of therapeutic interventions with potential risk for neurovascular tissues that other models do not allow. This study found that the insertion of a needle in the interphase of the fat and tendon from the medial side can avoid the penetration of neurovascular bundle, particularly if the procedure is US guided. In all the cadaveric samples, the sural nerve was parallel to the saphenous vein, at a mean distance of 5.2 mm lateral to the Achilles tendon to the height of the tendon body, about 5 cm from its distal insertion on the calcaneus. The distances observed in the current study in the cadaveric models and during the US-guided intervention were similar to those reported in previous studies, which supports the safety margin of the process [26,27]. However, anatomical variations in the course of this nerve are common. Anatomical studies of the nerve both corpse and live with US study describe a wide variability with different patterns of location [27,28], so the use of US guiding in therapeutic procedures such as percutaneous electrolysis is essential, since it will allow for the identification of structures and the application of the safest technique.

The percutaneous electrolysis approach described in this study could be effective in the treatment of pain in tendinopathies of the body of the Achilles tendon. Moderate evidence suggests a positive effect of US-guided percutaneous electrolysis treatment for tendon-related pain [20]. In addition, an animal study has found that the application of percutaneous electrolysis can help to release the nerve tissue from a fibrous entrapment [29]. The authors proposed that percutaneous electrolysis combines the mechanical effect of the needle and the galvanic current as a mechanism of connective tissue breakdown [29].

We hypothesize that the form of application of the percutaneous electrolysis with several paths at different angles from the medial aspect of the tendon at the interphase between the Achilles tendon and the Kager’s triangle could cause the rupture of the peritendinous adhesions and the neural tissue posterior to the tendon. The basis for performing this approach would be the same as that of minimally invasive surgery described and used by Mafulli [17]. Various studies have reported good results with this technique in pain in the AT of the tendon body, where with a strong suture introduced in the interphase, and making gentle movements on the rocker similar to the use of a saw in different directions, the neovascularization and neoinnervation present in the deep part of tendons with tendinopathy would be broken [17,18]. Both techniques, stripping and percutaneous electrolysis, would seek to eliminate the neoinnervation of the pathological portion of the tendon, while minimizing tissue damage.

Finally, some limitations of the study must be recognized. First, US imaging and anatomical dissection were conducted in a small number of individuals and specimens, respectively. No data for gender differences in needle placement were able to be collected. Similarly, the anthropometric data of the leg could influence the observed distances. Second, we used an anatomical reference point 5 cm from the distal insertion of the tendon as it is considered the most common zone for degeneration in tendinopathies of the tendon body. Consequently, data must be considered for this point of access to the tendon. Finally, all needle insertions were performed by an experienced clinician. We do not know the safety and accuracy of this needling procedure when applied by a novice clinician, or the reliability of either approach. Finally, it is important to note that the current study did not assess the effectiveness of the proposed intervention.

## 5. Conclusions

This validation study supports that percutaneous electrolysis can be safely performed for targeting the Kager’s fat–Achilles tendon interphase since penetration of the sural nerve was not observed in any needle approach when performed by an experienced clinician.

## Data Availability

Data are available upon reasonable request.
